# Linking elastographic evaluation benign and malignant liver lesion with histopathological data

**DOI:** 10.6026/973206300214885

**Published:** 2025-12-15

**Authors:** Mohd Ismail, Anima Ranjni Xalxo, Rajeev Kumar Ranjan, Suresh Kumar Toppo, Soumik Pal, Harish S Gupta, Manisha Oraon, Sonali Priyadarsini Reddy

**Affiliations:** 1 Department of Radio-diagnosis, Rajendra Institute of Medical Sciences, Ranchi, Jharkhand, India

**Keywords:** ARFI elastography, liver lesions, tissue stiffness, ultrasound, histopathological correlation, diagnostic accuracy

## Abstract

Differentiating benign from malignant focal liver lesions remains a significant diagnostic challenge with conventional imaging modalities. Acoustic Radiation Force Impulse (ARFI) elastography offers a non-invasive approach by quantitatively measuring tissue stiffness through shear wave velocity (SWV). ARFI elastography findings were compared with histopathology to assess diagnostic accuracy among 124 patients with focal liver lesions. Malignant lesions demonstrated significantly higher SWV values than benign lesions and a cutoff of 1.96 m/s achieved excellent sensitivity, specificity and overall accuracy. ARFI elastography is a highly reliable and superior adjunct to conventional ultrasound for non-invasive differentiation of benign and malignant liver lesions.

## Background:

Focal liver lesions are commonly encountered in clinical practice, with advances in imaging technology enabling detection of increasingly smaller lesions [1]. The differentiation between benign and malignant liver lesions is crucial for appropriate patient management, as it determines the need for further intervention, treatment strategies and prognosis [2]. Conventional ultrasound (US) is often the first-line imaging modality for liver evaluation due to its wide availability, non-invasive nature and cost-effectiveness [3]. However, conventional US have limitations in characterizing liver lesions, with overlapping imaging features between benign and malignant entities [4]. Ultrasound elastography has emerged as a promising technique for tissue characterization by assessing mechanical properties [5]. Among various elastographic techniques, Acoustic Radiation Force Impulse (ARFI) elastography provides quantitative measurement of tissue stiffness through shear wave velocity (SWV) assessment [6]. ARFI imaging utilizes short-duration acoustic radiation forces to generate localized tissue displacement, with the resulting shear wave propagation speed directly correlating with tissue stiffness [7]. This technique offers several advantages, including real-time B-mode image guidance, quantitative assessment and evaluation of deeper tissues without external compression [8]. Recent studies have demonstrated the utility of ARFI elastography in liver fibrosis assessment [9, 10]. However, its application in characterizing focal liver lesions remains an area of active investigation. Despite these advances, there remains a research gap regarding the optimal diagnostic thresholds and comprehensive histopathological correlation of ARFI elastography findings across diverse liver lesion types [11, 12, 13- 14]. Furthermore, the comparative diagnostic performance of ARFI elastography versus conventional ultrasound needs further elucidation in large prospective studies [15]. Therefore, it is of interest to evaluate the diagnostic potential of ARFI elastography as a non-invasive tool for differentiating between benign and malignant liver lesions by quantifying tissue stiffness, with histopathological correlation as the reference standard.

## Materials and Methods:

## Study design and setting:

A descriptive cross-sectional study, observational study was conducted over an 18-month period from January 2023 to June 2024 at the Department of Radiodiagnosis, Rajendra Institute of Medical Sciences, Ranchi, India. The study protocol was approved by the Institutional Ethics Committee (Memo No. 101 IEC, RIMS, dated 09/03/2024) and written informed consent was obtained from all participants prior to enrollment.

## Sample size calculation:

The sample size was calculated based on prevalence data from previous studies [16, 17]. With an estimated prevalence of focal liver lesions of 27%, 95% confidence interval and 8% margin of error, the required sample size was calculated to be 124 participants.

## Inclusion and Exclusion Criteria:

Inclusion criteria comprised: (1) patients with liver lesions detected via ultrasound imaging; (2) patients undergoing histopathological evaluation to determine the benign or malignant nature of liver lesions; (3) patients with complete clinical and demographic records suitable for analysis.

Exclusion criteria included: (1) patients with incomplete or unreliable ARFI elastography data; (2) patients with liver lesions previously subjected to treatment or biopsy prior to ARFI examination; (3) patients with unavailable or inconclusive histopathological findings; (4) patients with contraindications for ARFI or histopathological procedures; (5) patients with a history of liver resection or transplantation.

## Imaging protocol and equipment:

All participants underwent conventional ultrasound examination followed by ARFI elastography using a Philips Affinity 70 sonography system equipped with ElastPQ software. A C5-1 convex probe operating at Tissue Harmonic Imaging (THI; 3-5 MHz) was utilized for all examinations. Conventional ultrasound evaluation included assessment of lesion number, echogenicity, margins, posterior acoustic enhancement and color Doppler findings. Diagnostic criteria for benign and malignant lesions on conventional ultrasound were based on established guidelines [18, 19].

## ARFI elastography procedure:

ARFI elastography was performed with patients in supine or lateral decubitus positions during breath-holding (excluding deep inspiration). The depth of ARFI penetration was limited to 8 cm and measurements were taken at a minimum distance of 2 cm from the liver capsule. A 10x5 mm region of interest (ROI) was used to quantify tissue stiffness within both the focal masses and the surrounding liver parenchyma. Five separate measurements were acquired for each mass, while two measurements were obtained from the adjacent parenchyma. Regions exhibiting degeneration (necrotic, cystic, hemorrhagic, or calcified areas) and locations containing vascular or biliary structures were excluded from the ROI. The device-generated mean and standard deviation values for each mass and parenchyma were recorded for each participant.

## Histopathological examination:

Based on individual patient needs, recommendations were made for fine-needle aspiration cytology (FNAC), core biopsy, or excisional biopsy. Tissue samples obtained from these procedures were submitted for comprehensive histopathological examination, which served as the definitive diagnostic standard.

## Statistical analysis:

Data were compiled in Microsoft Office Excel 2007 and analyzed using SPSS version 15 and Epiinfo (3.5) software. Descriptive statistics were presented as mean ± standard deviation (SD) for continuous variables and as frequencies and percentages for categorical variables. Student's t-test and ANOVA were used for comparison of continuous variables between groups. Chi-square test was used for categorical variables. Receiver operating characteristic (ROC) curve analysis was performed to determine the optimal cutoff value for differentiating benign from malignant lesions. Sensitivity, specificity, positive predictive value (PPV), negative predictive value (NPV) and diagnostic accuracy were calculated. A p-value < 0.05 was considered statistically significant.

## Results:

The study included 124 patients with focal liver lesions, comprising 74 males (59.7%) and 50 females (40.3%). The mean age of participants was 51.3 ± 12.7 years. Among the 124 patients, 62 (50%) had benign lesions and 62 (50%) had malignant lesions based on histopathological examination. The distribution of lesion types is presented in Table 1 (see PDF) 0. Conventional ultrasound correctly identified 59 of 62 malignant lesions (sensitivity 95%) and 40 of 62 benign lesions (specificity 70%). The overall diagnostic accuracy of conventional ultrasound was 82.5%. Among the misdiagnosed cases, 3 malignant lesions (all metastases) were incorrectly classified as hemangiomas on conventional ultrasound. Conversely, 18 benign lesions were incorrectly identified as malignant, including 4 FNH misdiagnosed as adenomas, 4 hemangiomas misdiagnosed as metastases and 6 regenerating nodules misdiagnosed as HCC. The mean SWV values for different lesion types are presented in Table 2 (see PDF). Malignant lesions demonstrated significantly higher mean SWV values (2.66 ± 0.45 m/s) compared to benign lesions (1.53 ± 0.34 m/s) (p < 0.001). Among malignant lesions, cholangiocarcinoma showed the highest mean SWV (3.32 ± 0.22 m/s), followed by metastasis (2.99 ± 0.11 m/s) and HCC (2.27 ± 0.25 m/s). Among benign lesions, FNH had the highest mean SWV (1.87 ± 0.04 m/s), while adenoma had the lowest (1.24 ± 0.01 m/s). The mean SWV values of lesions and surrounding liver parenchyma are presented in Table 3 (see PDF). In patients with cirrhotic liver parenchyma (n=43), the mean parenchymal SWV was 2.85 ± 0.11 m/s, significantly higher than in non-cirrhotic livers (n=81) with mean SWV of 1.40 ± 0.12 m/s (p < 0.001). HCC lesions in cirrhotic livers showed lower mean SWV (2.27 ± 0.25 m/s) compared to the surrounding cirrhotic parenchyma (2.85 ± 0.11 m/s). In contrast, metastatic lesions demonstrated higher mean SWV (2.99 ± 0.11 m/s) compared to the surrounding non-cirrhotic parenchyma (1.40 ± 0.12 m/s). ROC curve analysis revealed an area under the curve (AUC) of 0.99 for ARFI elastography in differentiating benign from malignant liver lesions. The optimal cutoff value was determined to be 1.96 m/s, with sensitivity of 97%, specificity of 98%, PPV of 98%, NPV of 97% and diagnostic accuracy of 97.5%. At various cutoff values, the diagnostic performance of ARFI elastography is presented in Table 4 (see PDF).

## Discussion:

This study demonstrates that ARFI elastography is a highly accurate, non-invasive method for differentiating benign from malignant liver lesions, with significantly superior diagnostic performance compared to conventional ultrasound. Our findings revealed significant differences in tissue stiffness between benign and malignant lesions, with malignant lesions demonstrating consistently higher SWV values. The mean SWV values observed in our study are largely consistent with previous reports. The high fibrous content in cholangiocarcinoma likely contributes to increased tissue stiffness, making it readily distinguishable from other lesions using ARFI elastography [20, 21]. Similarly, metastatic lesions showed high mean SWV values (2.99 m/s), which may be explained by the fibrous stroma often associated with metastatic deposits [22, 23]. Interestingly, HCC lesions in our study demonstrated lower mean SWV values (2.27 m/s) compared to the surrounding cirrhotic parenchyma (2.85 m/s). This phenomenon may be attributed to the cellular architecture of HCCs, which typically consist of multiple cells arranged in cords with limited connective tissue, contrasted with the fibrotic nature of cirrhotic liver parenchyma [24]. Among benign lesions, FNH demonstrated the highest mean SWV (1.87 m/s), which aligns with the known high fibrous content characteristic of these lesions [25]. This finding could be clinically useful in differentiating FNH from other benign lesions, particularly hepatic adenomas, which showed the lowest mean SWV (1.24 m/s) in our study. The ability to distinguish between FNH and adenomas is clinically relevant, as FNH typically requires no treatment, while adenomas may necessitate surgical resection due to the risk of hemorrhage and malignant transformation [26, 27]. These differences may reflect variations in study populations, equipment and methodology. Nevertheless, the high AUC (0.99) in our study underscores the excellent discriminatory power of ARFI elastography for liver lesion characterization. The diagnostic performance of conventional ultrasound in our study (sensitivity 95%, specificity 70% and accuracy 82.5%) is consistent with previous reports [28]. The relatively low specificity of conventional ultrasound highlights the limitations of morphological assessment alone and underscores the potential value of adding ARFI elastography to the diagnostic armamentarium. The integration of ARFI elastography with conventional ultrasound could potentially reduce unnecessary biopsies and improve diagnostic confidence. Our study has several limitations. First, the sample size, while adequate for statistical analysis, was relatively small for subgroup analyses of less common lesions such as cholangiocarcinoma. Second, ARFI elastography has technical limitations, including depth restrictions and susceptibility to motion artifacts. Third, we did not evaluate inter-observer variability in ARFI measurements, which could affect reproducibility. Finally, our study was conducted at a single center, which may limit the generalizability of our findings. Future research should focus on larger multicenter studies to validate our findings, establish standardized protocols for ARFI elastography of liver lesions and evaluate the impact of this technique on clinical decision-making and patient outcomes. Additionally, studies comparing ARFI elastography with other advanced imaging modalities such as contrast-enhanced ultrasound, MRI and CT would be valuable.

## Conclusion:

ARFI elastography is a highly accurate, non-invasive method for differentiating benign from malignant liver lesions, with significantly superior diagnostic performance compared to conventional ultrasound. Using a cutoff value of 1.96 m/s, ARFI elastography demonstrated excellent sensitivity (97%), specificity (98%) and diagnostic accuracy (97.5%). The technique provides quantitative assessment of tissue stiffness that complements conventional ultrasound findings and this may reduce the need for invasive procedures. Thus, the integration of ARFI elastography into routine clinical practice for the characterization of focal liver lesions is needed for improving diagnostic confidence and patient management.
